# Buprenorphine versus Morphine in Paediatric Acute Pain: A Systematic Review and Meta-Analysis

**DOI:** 10.1155/2018/3792043

**Published:** 2018-08-07

**Authors:** Nathan Murray, Utsav Malla, Ruan Vlok, Alice Scott, Olivia Chua, Thomas Melhuish, Leigh White

**Affiliations:** ^1^Department of Anaesthesia and Pain Medicine, Sunshine Coast University Hospital, Birtinya, QLD, Australia; ^2^Wagga Wagga Base Hospital, Wagga Wagga, NSW, Australia; ^3^School of Medicine Sydney, University of Notre Dame Australia, Fremantle, NSW, Australia; ^4^School of Medicine, University of New South Wales, Sydney, NSW, Australia; ^5^Department of Intensive Care Medicine, Royal Prince Alfred Hospital, Sydney, NSW, Australia; ^6^School of Medicine, University of Queensland, Brisbane, QLD, Australia

## Abstract

**Introduction:**

In lab-based studies, buprenorphine appears to have a ceiling effect on respiratory depression but not on analgesia. There is increasing evidence in adult patients that buprenorphine has no ceiling effect on analgesia or side effects. The aim of this study was to investigate the efficacy and adverse effects of buprenorphine versus morphine in paediatric acute pain.

**Methods:**

A systematic review of five databases was performed until May 2018. Only randomised controlled trials were eligible for inclusion. The outcomes of interest included pain, respiratory depression, nausea, sedation, dizziness, and pruritus.

**Results:**

Four randomised controlled trials (*n*=195) were included. The only outcome measuring analgesic efficacy was time to breakthrough analgesia. Buprenorphine had a significant increase in time to breakthrough analgesia by 114.98 minutes compared to morphine (95% CI = 42.94 to 187.01; *I*^2^ = 0; *p*=0.002). There was no significant difference in the rates of adverse effects.

**Conclusions:**

Buprenorphine provided a longer duration of analgesia than morphine. This in combination with its unique sublingual preparation could prove particularly advantageous in the paediatric population. The studies included are likely underpowered to detect differences in the incidence of adverse effects; therefore, the same precautions should be taken as with any other opioid.

## 1. Introduction

Acute pain is a common adverse outcome experienced by the paediatric population, particularly in the postoperative period. This is often undertreated due to difficulties in reliably quantifying pain severity and fear of adverse effects from analgesia [1–3]. Poor management of postoperative pain is associated with a range of both short-term and long-term complications. Pain is associated with higher levels of anxiety, development of avoidance behaviour, and increased levels of both patient and parental distress [1]. Severe acute pain has been shown to lead to the development of chronic pain in up to twenty percent of children involved [4].

Effective management of acute pain typically involves a multimodal analgesic approach, with opioids a mainstay for moderate to severe pain [4, 5]. Common side effects of opioids include sedation, nausea, constipation, dizziness, and pruritus [1]. Respiratory depression is a troublesome and dose-limiting adverse effect associated with opioids that can limit their potential to provide adequate analgesia [5].

Buprenorphine is a semisynthetic, partial mu-opioid receptor agonist that has theoretical safety and efficacy advantages over pure opioid agonists, through its proposed ceiling effect on respiratory depression but not analgesic effect [4]. Whether this ceiling effect is reproduced in the paediatric population is controversial, with few studies demonstrating the safety and efficacy of buprenorphine in this population [4, 6].

This study aims at characterising the safety and efficacy of buprenorphine when used in the paediatric population for the management of acute pain by performing a systematic review and meta-analysis of randomised controlled trials comparing buprenorphine with morphine.

## 2. Methods

### 2.1. Search Strategy

A systematic search of five databases (PubMed, MEDLINE, Web of Science, Scopus, and CINAHL) was conducted. This was completed by two independent reviewers (L. W. and A. H.) from the time of inception of the databases until May 2018, searching the terms: (i) “buprenorphine” AND “post-operative pain”; (ii) “buprenorphine” AND “surgical pain”; and (iii) “buprenorphine” AND “acute pain.” For completeness, manual reference and citation checks of included papers were performed to identify and include relevant studies.

### 2.2. Inclusion Criteria

Included studies were required to report on the efficacy of buprenorphine versus morphine in the management of acute pain in the paediatric population, in the hospital setting. RCTs were the only study design eligible for inclusion. There were no language criteria for inclusion. Each study was independently assessed and agreed upon by two reviewers (L. W. and A. H.) before being included in this systematic review.

As morphine is a well studied and commonly prescribed opioid, this was chosen as a “treatment-as-usual” control group. This design was chosen to maximize the external validity of this review by ensuring the comparisons are in line with clinical decisions faced by those managing acute pain in a hospital setting. Studies that investigated the use of buprenorphine for the management of opioid dependence or chronic pain were excluded.

### 2.3. Data Extraction

Data were independently extracted from each article that met inclusion criteria by two reviewers (L. W. and A. H.). These data included the study design, subject characteristics, and clinical outcomes. The data extracted by each reviewer were compared for homogeneity.

### 2.4. Clinical Outcome Measures

The primary outcome of interest in this systematic review was analgesic effect, as measured by time to breakthrough analgesia. Secondary outcomes considered in this review were adverse effects of opioids including sedation, nausea, dizziness, pruritus, and respiratory depression.

### 2.5. Level of Evidence, Risk of Bias, and Outcome Level of Evidence Ranking

“*The Centre for Evidence-Based Medicine (CEBM) Levels of Evidence: Introductory Document*” was used to evaluate the included articles [7]. Methodological quality and risk of bias were assessed using the Cochrane Collaboration's tool for assessing risk of bias [8, 9].

### 2.6. Statistical Analyses

RevMan 5.3 software (Nordic Cochrane Centre, Copenhagen, Denmark) was used to analyse the combined data, using the weighted mean difference (WMD) with 95% confidence interval (CI) for continuous outcomes and the odds ratio (OR) with 95% CI for dichotomous outcomes. The Mantel–Haenszel random effects model was used. Heterogeneity was assessed using the *I*^2^ statistic, with an *I*^2^ > 50% indicating significant heterogeneity. A *p* value of <0.05 provided evidence of significant relative risk (RR) and WMD. A *p* value of <0.10 was used to demonstrate heterogeneity of intervention effects.

## 3. Results

### 3.1. Literature Search Results

**Figure 1:**
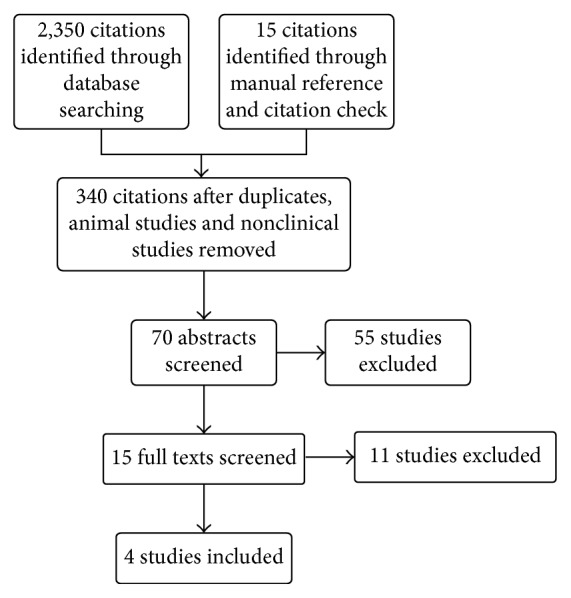
Study flow diagram.

**Figure 2:**
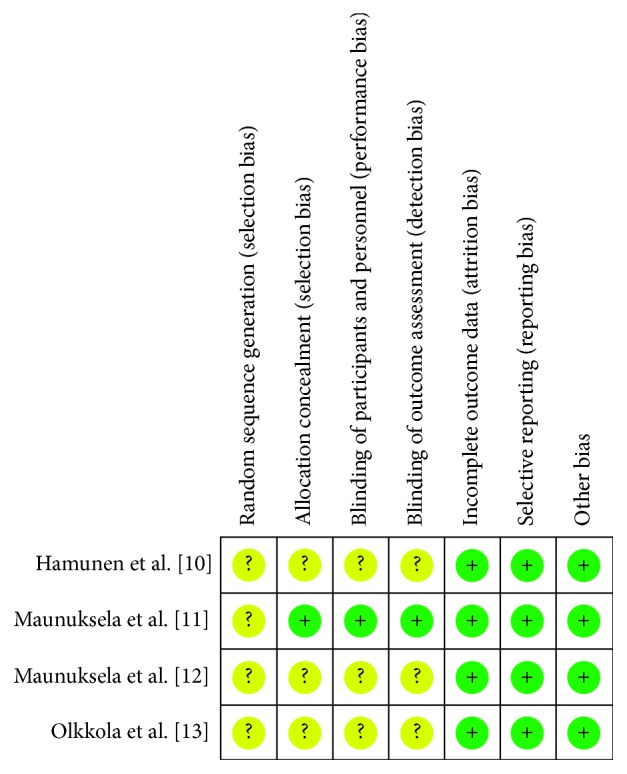
Risk of bias summary.

The initial systematic literature search yielded 2,350 citations, and a further 15 citations were identified through a manual citation and reference search of relevant articles (Figure 1). Following the removal of duplicates, animal studies, and nonclinical studies, 340 citations remained. Of these, 70 abstracts were screened and 15 full texts were retrieved for review. Four articles met the inclusion criteria (Figure 1). These four studies included 193 patients. All studies investigated the management of acute pain in paediatric patients in the hospital setting, comparing buprenorphine to morphine (Table 1). Each study was then screened for risk of bias and methodological quality using the Cochrane Collaboration's tool for assessing the risk of bias (Figure 2).One study was deemed to be a high-quality RCT, leaving three low-quality RCTs.

#### 3.1.1. Pain and Rescue Analgesia

**Figure 3:**
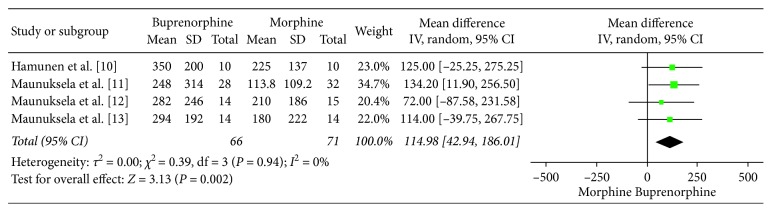
Time to breakthrough analgesia.

None of the studies retrieved reported pain scores. The only measure of analgesic effect was time to breakthrough analgesia which was significantly increased with the use of buprenorphine by 114.98 minutes (95% CI = 42.94 to 187.01; *I*^2^ = 0; *p*=0.002; Figure 3).

#### 3.1.2. Adverse Outcomes

Respiratory depression was measured by three studies, all with different outcome measures. Overall, there was no significant difference in respiratory depression with buprenorphine or morphine (OR = 5.57; 95% CI = 0.26 to 121.27; *p*=0.27). Nausea was measured by two studies with no significant difference (OR = 2.03; 95% CI = 0.69 to 5.93; *I*^2^ = 0%; *p*=0.20). There was no significant difference in the incidence of sedation, dizziness, or pruritus (*p* > 0.05).

## 4. Discussion

This is the first systematic review to investigate the effect of buprenorphine as an alternative to morphine for paediatric patients. This study included 4 RCTs, with 193 patients. Only the RCTs that aimed to compare buprenorphine to morphine were included in this review, in regard to their analgesic efficacy and side effects respective to paediatric acute pain.

The primary outcome of this study was time to breakthrough analgesia. Compared to morphine, buprenorphine resulted in a significantly increased time to breakthrough analgesia by 114.98 minutes (95% CI = 42.94 to 187.01; *I*^2^ = 0; *p*=0.002). The secondary outcomes in this study were adverse effects of buprenorphine versus morphine. One high-quality and two low-quality RCTs confirmed there were no significant differences in respiratory depression with buprenorphine and morphine [10–12]. All studies found no difference in nausea, sedation, dizziness, and pruritis [10–13]. This suggests that buprenorphine offers clinically significant analgesic benefits in the paediatric population and appears to have a similar adverse effect profile. Interestingly, buprenorphine is available in sublingual formulation, which may offer a noninvasive alternative to intravenous agents for acute paediatric pain.

The present meta-analysis includes a relatively small number of patients (*n*=193) limited to the postoperative patient. Additionally, in all four studies, pain is measured by the surrogate marker of time to breakthrough analgesia. This outcome may not necessarily be the most representative of onset of pain, as delay to administration may affect results. Further research is suggested by utilizing tools such as the Wong–Baker FACES Pain Rating Score [14]. Finally, given that these were small studies, it is unlikely that they were adequately powered to detect significant differences in adverse effect. The adverse effect rate is in keeping with larger adult based meta-analyses, and therefore, larger studies are unlikely to add to the results seen in this meta-analysis [15, 16].

In conclusion, buprenorphine provided a longer duration of analgesia than morphine. This in combination with its unique sublingual preparation could prove particularly advantageous in the paediatric population. Given that it had no difference in the incidence of adverse effects, the same precautions should be taken as with any other opioid.

## Figures and Tables

**Table 1:** Study characteristics.

Study	Number of patients (buprenorphine : morphine)	Mean age (buprenorphine : morphine)	Mean weight (buprenorphine : morphine) (kg unless stated otherwise)	Intervention	Setting	Outcomes
Hamunen et al. [10]	10 : 10	7.2 ± 1.2 : 6.8 ± 1.3	24.7 ± 5.0 : 22.1 ± 5.2	Intravenous morphine 100 mcg/kg, and intravenous buprenorphine 3.0 mcg/kg. Single dose	Ophthalmic surgery	(1) Respiratory depression (2) Nausea (3) Rescue analgesia (4) Hypotension (5) Time to analgesia

Maunuksela et al. [11]	28 : 32	10.4 ± 3.2 : 9.9 ± 2.9	33.7 ± 12.2 : 33.8 ± 11.8	Intravenous buprenorphine 6 mcg/kg or intravenous morphine 150 mcg/kg. Repeat dosing	Orthopaedic surgery	(1) Time to analgesia (2) Respiratory depression (3) Nausea (4) Sedation (5) Dizziness

Maunuksela et al. [12]	14 : 14 14 : 15	Buprenorphine 1.5 mcg/kg = 3.5 ± 2.3 : morphine 50 mcg/kg = 2.7 ± 1.9; buprenorphine 3.0 mcg/kg = 2.2 ± 1.2 : morphine 100 mcg/kg = 3.2 ± 2.3	Buprenorphine 1.5 mcg/kg = 13.8 ± 5.4 : morphine 50 mcg/kg = 12.9 ± 4.8; buprenorphine 3.0 mcg/kg = 10.7 ± 3.2 : morphine 100 mcg/kg = 13.2 ± 4.6	Buprenorphine 1.5 mcg/kg or morphine 50 mcg/kg; buprenorphine 3.0 mcg/kg or morphine 100 mcg/kg. All intravenous. Repeat dosing	Thoracotomy	(1) Time to analgesia (2) Respiratory depression (3) Nausea (4) Pruritus

Olkkola et al. [13]	28 : 28	Six months to six years	Not stated	Intravenous 50 or 100 mcg/kg morphine or intravenous 1.5 or 3.0 mcg/kg of buprenorphine. Repeat dosing	Lateral thoracotomy	(1) Respiratory depression

*Note*. Level of evidence assessed using “*The Centre for Evidence-Based Medicine (CEBM) Levels of Evidence: Introductory Document*” [7].
